# CT Radiomics Model for Predicting the Ki-67 Index of Lung Cancer： An Exploratory Study

**DOI:** 10.3389/fonc.2021.743490

**Published:** 2021-10-11

**Authors:** Qing Fu, Shun li Liu, Da peng Hao, Ya bin Hu, Xue jun Liu, Zaixian Zhang, Wen hong Wang, Xiao yan Tang, Chuan yu Zhang, Shi he Liu

**Affiliations:** The Affiliated Hospital of Qingdao University, Qingdao, China

**Keywords:** lung cancer, Ki-67, tomography, X ray, radiomics model, classification

## Abstract

**Objective:**

To establish a radiomics signature and a nomogram model based on enhanced CT images to predict the Ki-67 index of lung cancer.

**Methods:**

From January 2014 to December 2018, 282 patients with lung cancer who had undergone enhanced CT scans and Ki-67 examination within 2 weeks were retrospectively enrolled and analyzed. The clinical data of the patients were collected, such as age, sex, smoking history, maximum tumor diameter and serum tumor markers. Our primary cohort was randomly divided into a training group (n=197) and a validation group (n=85) at a 7:3 ratio. A Ki-67 index ≤ 40% indicated low expression, and a Ki-67 index > 40% indicated high expression. In total, 396 radiomics features were extracted using AK software. Feature reduction and selection were performed using the lasso regression model. Logistic regression analysis was used to establish a multivariate predictive model to identify high and low Ki-67 expression in lung cancer. A nomogram integrating the radiomics score was established based on multiple logistic regression analysis. Area under the curve (AUC) was used to evaluate the prediction efficiency of the radiomics signature and nomogram.

**Results:**

The AUC,sensitivity, specificity and accuracy of the radiomics signature in the training and validation groups were 0.88 (95% CI: 0.82~0.93),79.2%,84.3%,81.2% and 0.86 (95% CI: 0.78~0.94),74.6%,88.1%,79.8%, respectively. A nomogram combining radiomics features and clinical risk factors (smoking history and NSE) was developed. The AUC, sensitivity, specificity and accuracy were 0.87 (95% CI: 0.80~0.95), 75.0%, 90.2% and 83.5% in the validation group, respectively.

**Conclusion:**

The radiomics signature and nomogram based on enhanced CT images provide a way to predict the Ki-67 expression level in lung cancer.

## Introduction

Lung cancer is one of the most common malignant tumors that endangers human health and life, ranking first in the number of cancer-related deaths (1, 2). The proliferation mode and speed of tumor cells are related to the malignancy and prognosis of lung cancer (3–5). Ki-67 is a nuclear antigen expressed by cells in the proliferation phase that accurately reflect the proliferation activity of cells. Because of its short half-life, Ki-67 is significantly better than those proliferating cell nuclear antigens with a long half-life in evaluating the proliferative activity of tumor (6–9).

Presently, Ki-67 expression in lung cancer must be determined by biopsy or surgical histopathology, but biopsy samples generally represent only a small part of the tumor tissue. Because of the heterogeneous expression of Ki-67 in tumors, the Ki-67 index obtained by needle biopsy samples cannot fully and accurately represent the Ki-67 level of the entire tumor. This situation leads to deviations in results and even misdiagnosis and nonoptimal clinical decision-making (10). As a new research field, radiomics has obvious advantages in assessing tumor heterogeneity. It can discover and analyze different cell phenotypes of tumors (11–13) and provide comprehensive and quantitative tumor measurements, including texture, intensity, heterogeneity and morphological information, enabling a comprehensive analysis of the tumor phenotype (14–16). Zhou B et al. (17) found that twelve CT radiomic features were significantly associated with the Ki-67 of lung cancer, but they did not build a predictive model. Gu Q et al. (18) built a machine learning-based radiomics classifier to predict the Ki-67 index of non-small cell lung cancer, however, their study did not include cases with small cell lung cancer, so the model was not applicable to all patients with lung cancer. Moreover, these past studies have not established a nomogram model that combines radiomic features with clinical parameters, which may have better prediction efficiency. This study aimed to establish a radiomics signature based on enhanced CT images and a nomogram based on radscores and clinical parameters to predict the Ki-67 index of lung cancer.

## Materials and Methods

### Data Cohort

This retrospective study was approved by the Institutional Review Board. The data of 2286 consecutive patients with lung cancer confirmed by surgery between January 2014 and December 2018 were identified for this retrospective study.

The inclusion criteria were as follows: (1) a diagnosis of lung cancer by surgical pathological specimens and immuno-histochemical Ki-67 examination and (2) dual-phase enhanced chest CT examination before surgery.

The exclusion criteria were as follows: (1) no Ki-67 immunohistochemistry or enhanced CT examination at our hospital (n=1280); (2) poor image quality or image layer thickness greater than or equal to 5 mm (n=130); (3) incomplete clinical data (n=335); (4) prior neoadjuvant treatment before surgery (n=91); (5) small lesions (long diameter < 1 cm) (n=104); (6) other primary malignancies in the same period (n=64).

**Figure 1:**
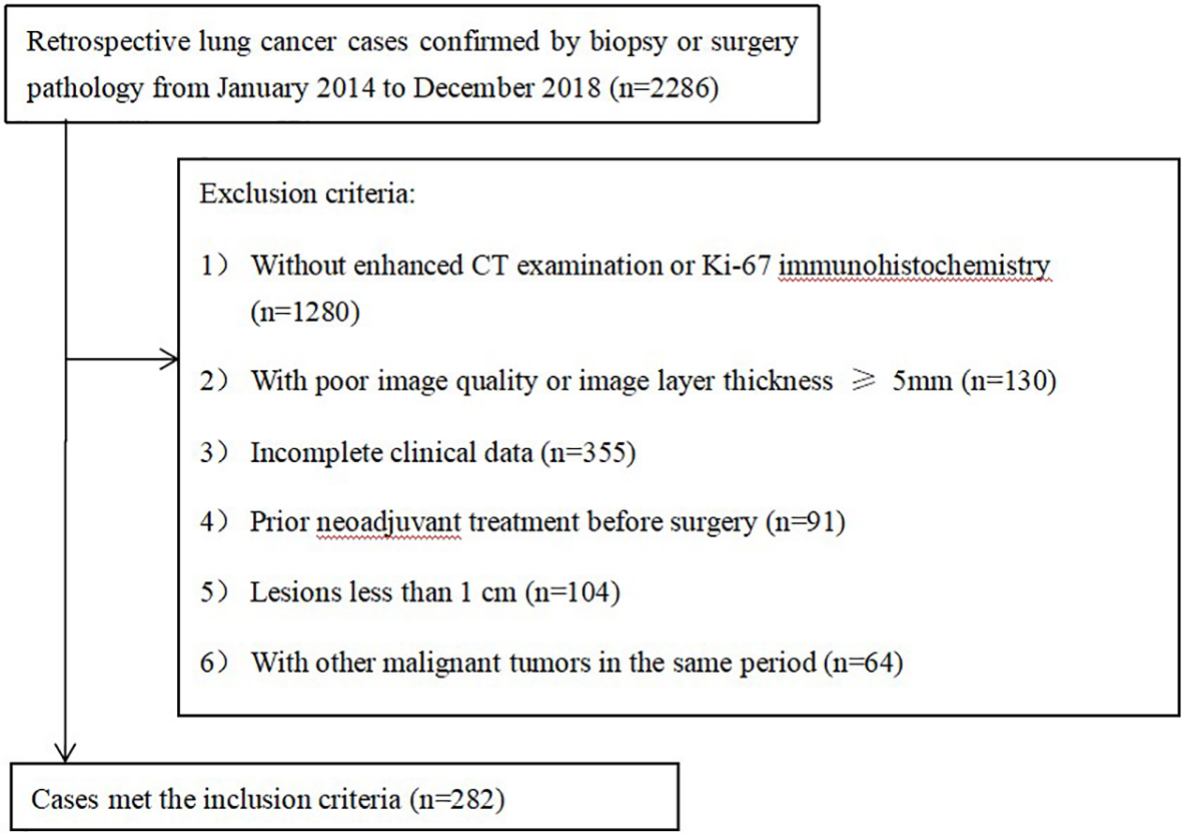
Flow diagram of the patient selection.

Two hundred eighty-two patients (178 men and 104 women with a mean age of 62.0 ± 8.9 years) were enrolled in our study (**Figure 1**), 158 patients with adenocarcinoma (ACC), 69 with squamous cell carcinoma (SCC), and 55 with neuroendocrine carcinoma (NEC) (including 38 patients with small cell lung cancer, 13 patients with large cell lung cancer, and 4 patients with carcinoid cancer). Using a stratified random sampling method, the patients were divided into a training group and a validation group at a ratio of 7:3.

The following information of the enrolled patients was evaluated: smoking history, sex, age, maximum tumor diameter, pathological type, and serum tumor markers, including neuron-specific enolase (NSE) serum, gastrin-releasing peptide precursor (ProGRP), carcinoembryonic antigen (CEA), squamous cell carcinoma antigen (SCCA) and cytokeratin 19 fragment (cYFRA21-1). Smoking history was defined as smoking for more than one year and smoking more than 20 cigarettes per day on average. Histological classification was based on H&E staining according to the WHO classification of malignant lung tumors.

### CT Protocol

All patients were scanned using a SOMATOM (Siemens Medical Systems, Germany) scanner or Brilliance iCT 256 (Philips Healthcare, Netherlands) scanner. The scanning parameters were as follows: tube voltage: 120 kVp; pixel size: 512×512; detector collimation: 64×0.6 and 128×0.625 mm; slice thickness: 5 mm; and reconstructed section thickness: 1 mm. Contrast-enhanced CT images were obtained by intravenous injection of 1.0 ml/kg of contrast material (iohexol injection; 300 mg/ml; Beijing, China) at a rate of 3.0-3.5 ml, followed by a saline flush (20 mL). CT images were acquired at 25 seconds and 70 seconds after the start of contrast medium injection, corresponding to the arterial and venous phases, respectively.

### Ki-67 Expression Measurement

Formalin-fixed, paraffin-embedded tissue sections with a thickness of 4 µm were created. The sections were then dried, dewaxed with xylene, rinsed in graded ethanol and rehydrated in double-distilled water. Immunohistochemistry (IHC) staining was performed using a Ki-67 protein antibody (Santa Cruz Biotechnology, California, USA) diluted 1:100. Cells with brown nuclei were considered positive.

The whole specimen was scanned, and positive cells in five areas with the highest positive density were selected, then a percentage of positively labeled cells were determined by counting more than 1000 tumor nuclei at 400 magnification. Because the most active part of tumor proliferation can represent the degree of tumor malignancy and affect the prognosis of patients. So according to previous relevant studies (19–21), the Ki-67 index in this study was the average value of the five areas with the highest percentage of Ki-67-labeled cells, and according to previous studies (22), low Ki-67 expression was defined as ≤ 40% positive staining, while over 40% positive staining was defined as high Ki-67 expression.

### Image Normalization and Feature Extraction

**Figure 2:**
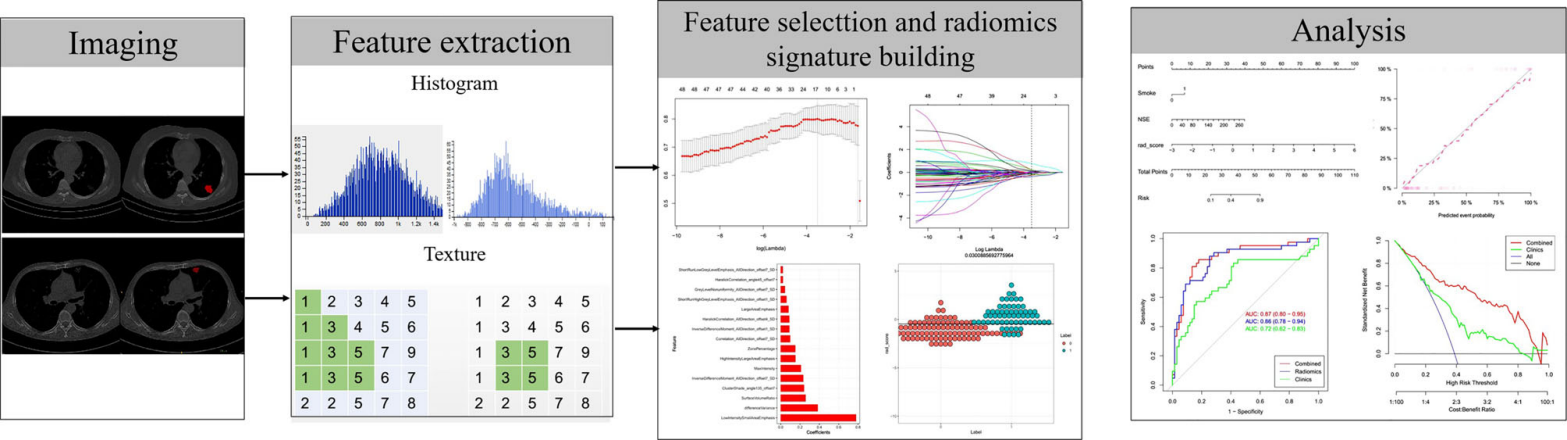
Flowchart of radiomics implementation in this study.

The workflow of radiomics implementation is displayed in **Figure 2**. All the images were normalized by z-score transformation, with intensity ranges for each imaging modality across all subjects with a mean of 0 and a standard deviation of 1.ITK-SNAP software (http://www.itksnap.org, version: 3.8.0, USA) was used to outline the lesion on the CT image with the largest diameter of the lesion. All lesion ROI outlining was completed by two radiologists with 12 years (HYB) and 10 years (SLL) of chest CT diagnosis experience, and the intragroup correlation coefficient (ICC) between among the observers was calculated. The ROI was outlined by the HYB once, and the second ROI was performed after a week to assess the observer’s ICC. SLL only performed the ROI once to evaluate the ICC between this physician and HYB. ICC>0.75 considers that the consistency is good. Both radiologists were blinded to the patient’s clinicopathological information. Commercial software (Analysis Toolkit 1.0.3; GE Healthcare, USA) was used to extract features. In total, 396 quantized features were extracted, such as 9 form factor features, 10 Haralick features, 11 gray level size zone matrix (GLSZM) features, 42 histogram features, 48 gray level cooccurrence matrix (GLCM) features with an offset of 1/4/7, and 60 gray level run-length matrix (GLRLM) features with an offset of 1/4/7.

### Development of the Radiomics Signature, Clinical Model, Radiomics Nomogram

To minimize overfitting, the least absolute shrinkage and selection operator (lasso) regression method was used to select the most valuable features from the primary datasets, and then validated in the validation cohort. The linear combination of selected features was used to calculate the radiomic scores (Rad-scores) for each patient. For validation, we evaluated the difference of rad score between the training set and the verification set, and calculated the sample size of the verification set using the method of “comparing the mean between the two groups”, which meets the statistical power of more than 0.8. The cut-off value obtained from the training set was used to calculate the metrics of the validation set.The predictive accuracy of the radiomics signature was quantified by the area under the receiver operating characteristic (ROC) curve (AUC) in both the training and validation sets.

Univariate logistic regression was used to select clinical risk factors for high Ki-67 lung cancer. The clinical features and imaging omics features with P<0.05 were used to develop a predictive model to distinguish low Ki-67 lung cancer from high Ki-67 lung cancer using multivariate logistic regression in the primary cohort. In logistic regression, backward stepwise selection was applied using a likelihood ratio test with Akaike’s information criterion as the stopping rule.

To provide clinicians with a quantitative tool to predict the Ki-67 level of lung cancer, a radiomics nomogram was established based on multivariate logistic analysis in the primary cohort. The algorithm built by the training set was used to calculate the Rad score in the validation set.

### Validation and Assessment of the Radiomics Nomogram

We assessed the value of the radiomics nomograms in training (n=197) and validation (n=85) data sets, including identification, calibration, and clinical value, and quantified the differential performance of AUC. The Hosmer-Lemeshow test was used together with the calibration curve to determine the goodness-of-fit of the nomogram. The validation data set was used to test the internal value of the radiomics nomogram.

Decision curve analysis (DCA) was used to calculate the net benefit of the threshold probability range in the training and validation data sets to estimate whether the nomogram was sufficiently reliable for clinical use. The net benefit was determined by calculating the difference between the true positive rate and weighted false positive rate of different threshold probabilities in the validation set. A “decision curve” was then drawn based on the threshold probability.

### Statistical Analysis

R statistical software (http://www.Rproject.org, version 3.4.4) was used for statistical analysis. Lasso regression was performed using the “glmnet” package. The “RMS” package was used to construct multivariate logistic regression, nomogram and calibration charts. DCA was performed using the “DCA. R” function. ROC curves were drawn and analyzed using the “proc” package. The Kolmogorov-Smirnov test was used to test the normality of the quantitative data, and the measurement data conforming to the normal distribution were expressed as x ± s. Counting data was expressed in frequency. Chi-squared test or Fisher’s exact test was used to compare the count data between groups, and independent samples t test was used to compare the measurement data. P<0.05 indicated a statistically significant difference.

## Results

### Comparison of the Clinical Data Results of the Training and Validation Groups and the Low Ki-67 and High Ki-67 Lung Cancer Groups

No significant differences were found in age, sex, the tumor diameter or pathological type between the training and validation groups (P>0.05; **Table 1**). Statistically significant differences were found in sex, age, and the pathological type between the high and low Ki-67 expression groups (P<0.05) (**Table 2**). High Ki-67 expression was more common in men, elderly individuals, and SCC patients.

**Table 1:** Comparison of the clinical data and pathological staging results of patients in the training and validation groups.

Clinical feature		Training group (n = 197)	Verification group (n = 85)	p value	t value or χ2 value
Sex	Male	126 (64.0%)	52 (61.2%)	0.66	0.20
	Female	71 (36.0%)	33 (38.8%)		
Age (years)		61.6 ± 8.9	62.4 ± 9.2	0.49	0.69
Tumor maximum diameter (cm)		4.1 ± 2.2	4.0 ± 2.3	0.58	0.55
Smoking	Yes	111 (56.3%)	41 (48.2%)	0.18	1.81
	No	86 (43.7%)	44 (51.8%)		
Pathological type	ACC	110 (55.8%)	48 (56.5%)	0.91	0.19*
	SCC	48 (24.4%)	21 (24.7%)		
	NEC	39 (19.8%)	16 (18.8%)		

*χ2 value (continuous variables were analyzed by t test, and categorical variables were analyzed by chi-square test).

**Table 2:** Comparison of the clinical data results between the low Ki-67 and high Ki-67 lung cancer groups.

Clinical feature		low Ki group (n = 175)	high Ki group (n =1 07)	p value	t value or χ2 value
Age (years)		62.2 ± 8.8	61.8 ± 9.1	0.74	0.33
Sex	Male	93 (53.1%)	85 (79.4%)	<0.01	19.73
	Female	82 (46.9%)	22 (20.6%)		
Smoking	Yes	76 (43.4%)	76 (71.0%)	<0.01	20.36
	No	99 (56.6%)	31 (29.0%)		
Tumor diameter (cm)		3.7 ± 2.0	4.6 ± 2.4	<0.01	-3.46
Pathological type	ACC	135 (77.1%)	23 (21.5%)	<0.01	89.77
	SCC	29 (16.6%)	40 (37.4%)		
	NEC	11 (6.3%)	44 (41.1%)		

Continuous variables were analyzed by t test, and categorical variables were analyzed by χ2 test. *u value: the overall variance of the two groups of data was uneven, and the rank-sum test was performed.

### Extraction/Selection of Radiomics Features and Construction of the Radiomics Signature

**Figure 3:**
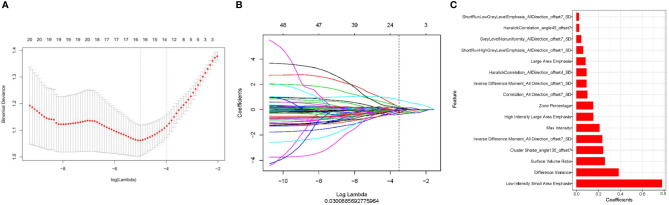
Use of lasso logistic regression to select features. **(A)** Binomial deviation versus parameter λ. Least absolute shrinkage and selection operator (LASSO) regression was used to screen the radiomic features, and cross-validation was used to select the optimal model parameter λ. The vertical axis is the binomial deviation, and the horizontal axis is the log (λ) value. λ, which represents the smallest binomial deviation of the model, is the optimal value (vertical dashed line). **(B)** Graph of the variation of the imaging omics feature coefficient with λ. The number above indicates the number of filtered features. **(C)** Screened 16 radiomics features and their weights.

**Figure 4:**
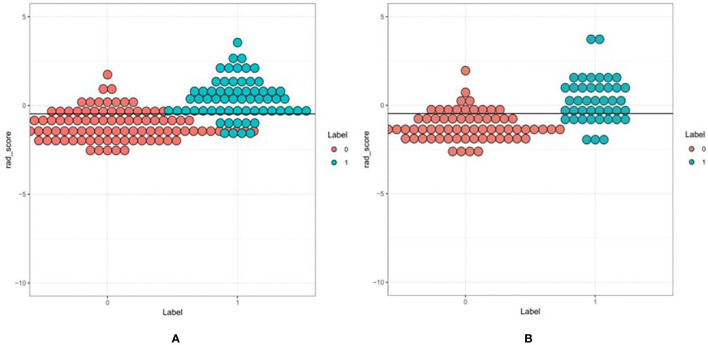
A set of verified rad scores in the training set **(A)** and validation set **(B)**. Red and green represent the true classification: the complete separation of red and green indicates that the radiomics rad-score can be classified well.

First, we performed repeatability evaluation (between and within data sets with a consistency coefficient> 0.75), and then removed highly correlated features (correlation coefficient> 0.6). Finally, we used lasso logistic regression to screen out 16 features (**Figures 3A**–**C**),including Low Intensity Small Area Emphasis, difference Variance, Surface Volume Ratio, Cluster Shade_angle135_offset7, Inverse Difference Moment_All Direction_offset7_SD, Max Intensity, High Intensity Large Area Emphasis, Zone Percentage,Correlation_All Direction_offset7_SD, Inverse Difference Moment_All Direction_offset1_SD, Haralick Correlation_All Direction_offset4_SD, Large Area Emphasis, Short Run High Grey Level Emphasis_All Direction_offset1_SD, Grey Level Non uniformity_All Direction_offset7_SD, Haralick Correlation_angle45_offset7, Short Run Low Grey Level Emphasis_All Direction_offset7_SD.The Rad-scores of each patient in the training and validation sets are shown in **Figures 4A, B**.

### Predictive Efficacy of the Imaging Radiomics Signature

**Figure 5:**
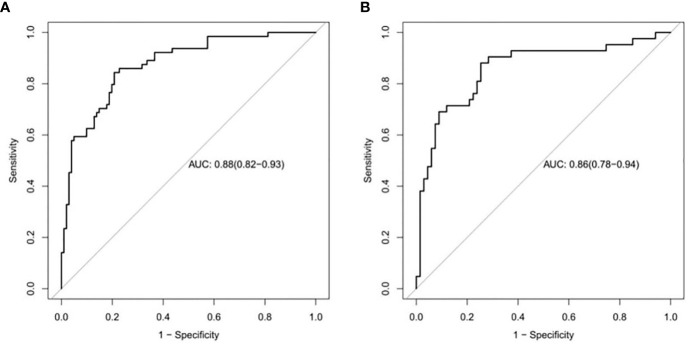
ROC curves to distinguish low Ki-67 lung cancer from high Ki-67 lung cancer based on the CT imaging model prediction model. The AUC in the training set was 0.88. **(A)**, and that in the validation set was 0.86 **(B)**.

The ROC curves of the training and validation groups are shown in **Figures 5A, B**. The AUC, accuracy sensitivity, specificity, positive predictive value, and negative predictive value were 0.88 (95% CI: 0.82~0.93), 81.2%, 79.8%, 84.4%, 88.9%, and 72% in the training group and 0.86 (95% CI: 0.78~0.94), 79.8%, 74.6%, 88.1%, 90.9%, and 68.5% in the validation group, respectively.

### Establishment of a Nomogram Combining Radiomics With Clinical Risk Factors

**Figure 6:**
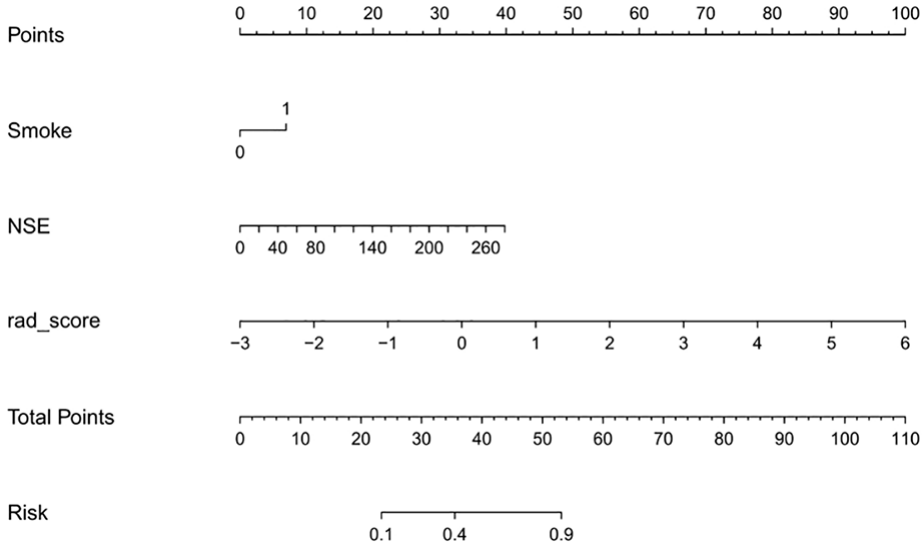
Nomogram used to distinguish between high and low Ki-67 expression levels in lung cancer.

Univariate analysis showed that the clinical factors were significantly related to the classification of low Ki-67 lung cancer and high Ki-67 lung cancer (**Table 3**). They include serum NSE and smoking (P < 0.05). The results of multivariate logistic regression analysis suggested that smoking, serum NSE and the rad score were independent predictors of low and high Ki-67 lung cancer classification (**Table 4**). A radiomics nomogram incorporating the predictive factors (including smoking, NSE, and the Rad score) was constructed (**Figure 6**).

**Table 3:** Positive results of univariate analysis for the classification of low and high Ki-67 lung cancer.

Variable	OR (95% CI)	P-value
Smoking	3.45 (1.78-6.88)	<0.01
Serum	1.00 (1.00-1.00)	0.02
NSE	1.05 (1.02-1.09)	<0.01

**Table 4:** Positive results of multivariate logistic regression analysis for the classification of low and high Ki-67 lung cancer.

Variable	OR (95% CI)	P-value
(Intercept)	0.48 (0.17-1.30)	0.16
Smoking	2.78 (1.17-6.90)	0.02
NSE	1.02 (0.99-1.06)	0.25
rad_score	5.16 (3.09-9.50)	<0.01

**Figure 7:**
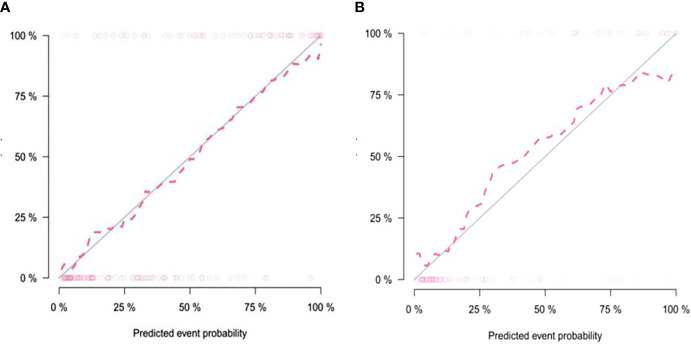
Calibration curve of the nomogram in the training group **(A)** and validation group **(B)**. The solid diagonal line represents the perfect prediction of the ideal model, and the dashed pink line represents the performance of the model. Closer plots of the two lines indicate that the prediction results are in good agreement with the pathological results, and the prediction ability is better.

**Figure 8:**
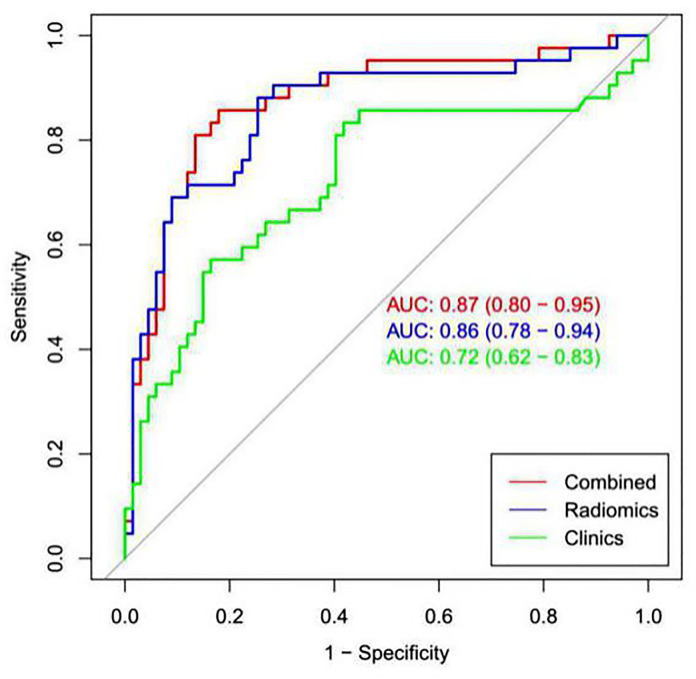
ROC analysis was used to compare the prediction efficiency among the nomogram, radiomics signature and clinical models. The red line shows the nomogram with AUC=0.87, indicating that the radiomics nomogram had better predictive performance than the clinical model (green line; AUC=0.72) or radiomics signature (blue line; AUC= 0.86).

**Figure 9:**
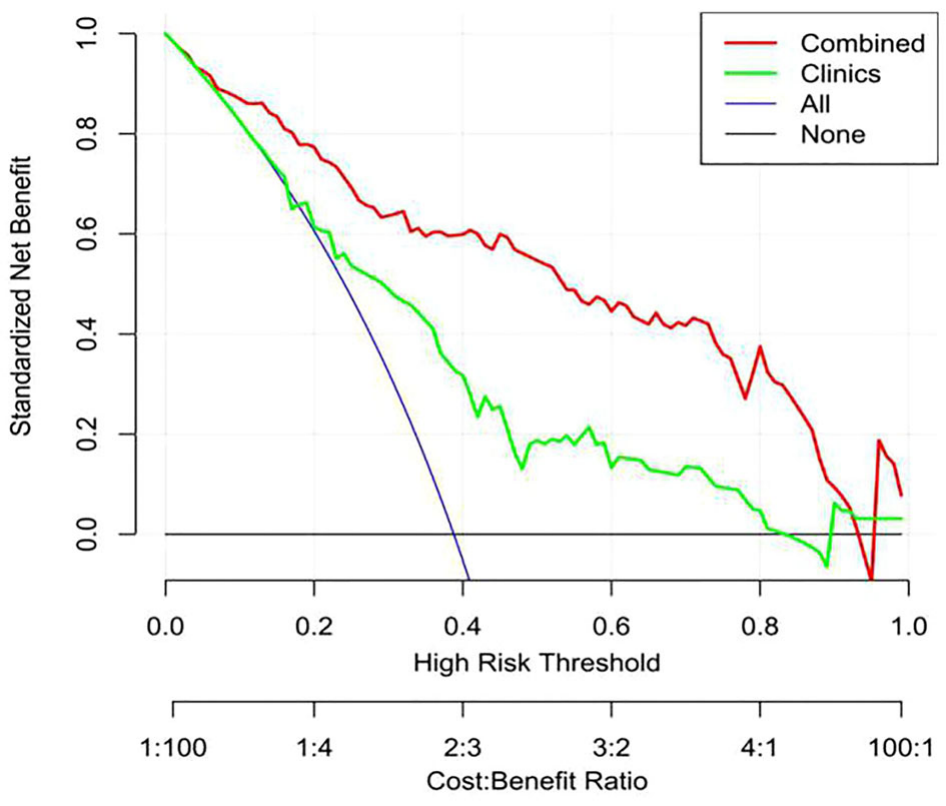
Decision curve analysis (DCA) of the nomogram. The y-axis shows net income, the red line represents the radiomics nomogram, the blue line represents the hypothesis that all patients have high Ki-67 expression, and the black line represents the hypothesis that no patient has high Ki-67 expression. The x-axis shows the threshold probability—that is, the expected benefit of the number of treatments equals the expected benefit of not receiving treatment. The decision curve shows that when the threshold probability is between 0.1 and 1, using a radiomics nomogram to predict Ki-67 expression is more beneficial than treating all patients or not treating patients.

The calibration curve showed that the predicted probability of the nomogram was consistent with the pathological findings (**Figure 7**). The results in **Table 5** and **Figure 8** show that the nomogram had better prediction efficiency than the radiomics signature and clinical model. The AUC value of the nomogram in the validation set was 0.87 (95% CI: 0.80-0.95), the accuracy was 0.83 (95% CI: 0.75-0.90), the sensitivity was 75.0%, and the specificity was 90.2%. **Figure 9** shows the DCA of the radiomics nomogram. When the threshold probability is in the range of 0.1–1.0, the radiomics nomogram is superior to the model of “all treatment” and “no treatment” strategies.

**Table 5:** Predictive ability of the radiomics nomogram, radiomics signature, and clinical model for the classification of low and high Ki-67 lung cancer.

Variable		AUC	(95% CI)	Accuracy	Sensitivity	Specificity
Clinical model	Train	0.77	(0.69~0.85)	0.69	0.59	0.82
	Test	0.72	(0.62~0.83)	0.66	0.54	0.80
Radiomics signature	Train	0.88	(0.82~0.93)	0.81	0.79	0.84
	Test	0.86	(0.78~0.94)	0.80	0.75	0.88
Radiomics nomogram	Train	0.91	(0.85~0.98)	0.83	0.79	0.85
	Test	0.87	(0.80~0.95)	0.83	0.75	0.90

## Discussion

Ki-67 nuclear protein is a marker of cell cycle and proliferation (9, 23, 24) and is typically used to estimate the population of proliferating cells. In malignant tumors, the percentage of Ki-67-positive cells is related to tumor invasion or tumor progression. Presently, the Ki-67 proliferation index is considered a tumor biomarker that is valuable for tumor diagnosis, treatment and prognosis (4, 24, 25). Previous studies on the CT texture features of non-small cell lung cancer (10, 26, 27) have shown that tumors have internal heterogeneity due to different biological behaviors and metabolic levels, and CT texture analysis can quantify tumor heterogeneity. Lung cancer is a highly heterogeneous tumor, and the heterogeneity of Ki-67 expression also exists in lung cancer. The Ki-67 labeling index can range from 1% to 90% in different intratumoral regions (28). High and low Ki-67 expression results in heterogeneity in the tumor cell proliferation rate, cell differentiation and subclonal region composition. Radiomics is a revolution to the traditional visual image features. It obtains high-throughput data and extracts a large number of quantitative features from the image through computer learning software to mine the quantitative information of the shape, texture and heterogeneity of the tumor itself, and screen the most valuable radiomic features to establish a prediction model (18, 29, 30). Radiomics can not only reduce the pain of patients undergoing biopsy, but also improve work efficiency and reduce the cost of patients. Therefore, predicting the expression of Ki-67 by analyzing the CT images of lung cancer is clinically significant.

The present study first used quantitative imaging histology, and then, based on CT images routinely used to diagnose tumors clinically, quantitative image texture analysis was used to estimate Ki-67 expression in lung cancer patients. The radiomics signature was an independent predictor of the expression status of Ki-67 in lung cancer and can distinguish between low Ki-67 lung cancer and high Ki-67 lung cancer well. The AUC of the validation group reached 0.86, and the accuracy, sensitivity, and specificity were 0.80, 0.75, and 0.88, respectively. Radiomics is expected to provide a noninvasive, convenient, and reproducible method to predict the Ki-67 expression status in lung cancer.

In the present study, the training and validation group showed differences in sex, the maximum tumor diameter, smoking status, and pathological type. High Ki-67 expression is more common in men, smokers, and patients with large lesions, SCC and small cell lung cancer. This finding is consistent with previous reports (31, 32). To predict the Ki-67 index, the AUC value of the clinical prediction model established in this study was only 0.72, while that of the radiomics signature was 0.86, much higher than that of the clinical model, indicating that the radiomics signature was significantly better than the simple clinical data prediction model in predicting the Ki-67 index of lung cancer. This study also established a nomogram prediction model combining the radiomics signature and clinically related factors. The data revealed that the AUC value of the validation group was 0.87, which was slightly higher than the predictive power of the radiomics signature alone (AUC=0.86) but significantly higher than the predictive power of the clinical model (AUC=0.72), and the prediction accuracy and specificity of the nomogram were improved. The prediction efficiency of the nomogram was better than that of the clinical model and radiomics signature model. A certain complementarity exists between the radiomics signature model and clinical model, but it is not obvious. The subjects of this study covered all pathological subtypes of lung cancer, so our model had better universality. Moreover, the results of this study showed that our model was more effective in predicting the Ki 67 index of lung cancer than the models built in previous studies (17, 18).Our model may become an accurate and noninvasive method to predict the status of Ki-67 in patients with lung cancer.

This study also has some limitations. First, this is a retrospective study with potential selection bias. Second, the sample size of this study was still relatively small, and the predictive ability of radiomics for the Ki-67 index of lung cancer must be further verified in a large sample. Third, although this study included patients with different pathological types of lung cancer, it did not specifically analyze the prediction of the Ki-67 index in a specific pathological type of lung cancer by radiomics. Different cutoff values of the Ki-67 index may need to be established for different pathological types of lung cancer, but the sample size of this study was not sufficiently large to perform this analysis. Therefore, this study is a preliminary exploratory study on the relationship between imaging features and the Ki-67 index of lung cancer. We will expand the sample size and integrate more clinical information to improve the performance and universality of the radiomics model.

In conclusion, we developed and validated the first nomogram model with good diagnostic performance for the classification of low Ki-67 lung cancer and high Ki-67 lung cancer based on the radiomics signature and clinical factors.

## Data Availability Statement

The original contributions presented in the study are included in the article/supplementary material. Further inquiries can be directed to the corresponding authors.

## Ethics Statement

The studies involving human participants were reviewed and approved by The Institutional Review Board of the Affiliated Hospital of Qingdao University. Written informed consent to participate in this study was provided by the participants’ legal guardian/next of kin. Written informed consent was obtained from the individual(s), and minor(s)’ legal guardian/next of kin, for the publication of any potentially identifiable images or data included in this article.

## Author Contributions

QF, SHL, and SLL conceived of the project, analyzed the data,and wrote the paper. YBH, ZXZ, WHW and XYT participated in data collection and processing. DPH, XJL and CYZ provided expert guidance, and reviewed the manuscript. All authors contributed to the article and approved the submitted version.

## Conflict of Interest

The authors declare that the research was conducted in the absence of any commercial or financial relationships that could be construed as a potential conflict of interest.

## Publisher’s Note

All claims expressed in this article are solely those of the authors and do not necessarily represent those of their affiliated organizations, or those of the publisher, the editors and the reviewers. Any product that may be evaluated in this article, or claim that may be made by its manufacturer, is not guaranteed or endorsed by the publisher.
